# Supplementary material for: Botched Ebola Vaccine Trials in Ghana: An Analysis of Discourses in the Media
Source: Vaccines (Basel). 2021 Feb 19;9(2):177. doi: 10.3390/vaccines9020177 (PMC7922854; doi:10.3390/vaccines9020177)
Supplement: Supplementary file 1 [file vaccines-09-00177-s001.zip › vaccines-1079242-supplementary.pdf]

Table 1.
Sample coding and analysis process

| Examples of content | Open coding | Axial coding | Theme building stage |
|---|---|---|---|
| We [media] weren't told that this thing was going on- | Media not knowing | Moral expectation to inform the public | **Moral panic discourses** |
| Parliamentarians didn't even know about it until we raised the red flag | Political leaders not knowing | | - Moral blunder discourses |
| We have never had a confirmed case [of Ebola] in Ghana; it has always been suspected so even if you are doing some trials, at least let us know how it is going to go | General public not knowing | Moral expectation to inform the public | |
| Because we don't know they usually use human being in trials … | Not knowing about human trials | Moral responsibility to educate the public | - Moral responsibility discourses |
| It all started when a parliamentary candidate blew the story to a journalist who did not know how to handle it. | Journalist not knowing good journalism | | |
| Let's be open minded of this experiments. People must know exactly what is all about. | General public needing to know | | |
| Prof. Binka and Prof. Koram are both Epidemiologist and not Virologist but they want to carry on with an Ebola clinical trials in Ghana. These are just selfish physicians wanting to use fellow Ghanaians as Guinea pigs for their personal interests | Selfish physicians using Ghanaians | Folk devils; Stigmatizing individuals | - Moral deviants |
| User 45: Big Western Pharmaceuticals want to use Ghanaians and Africans as cheap TEST-PIGS! They don't care. It's Ghanaians and Africans who die! | Big western pharmaceuticals using Ghanaians | Folk devils; Stigmatizing institutions | |

| | | | |
|---|---|---|---|
| Shame to the FDA and Ministry of Health officials involved in this God forsaken Ebola trials<br><br>So now our leaders want to use us like laboratory rats? God save us and punish our wicked leaders involved<br><br>Poverty has turned my people into guinea pigs. I can confidently say that some big man has received a big cut (thus big money).<br><br>And how can you give yourself out for 200Gh and phone? All in the name of Ebola vaccination and the so-called other things. | Shame on FDA and MoH<br><br>Leaders as irresponsible<br><br>Poverty has turned people into guinea pigs; Selfish leaders<br><br>Giving yourself for pittance | Stigmatizing volunteers | Stigmatizing discourses |
| User 88: This vaccine is very new and so how can they be sure that it's safe and people won't end up developing the Ebola virus?<br><br>Just listen what the man was saying. They have tried the vaccine on animals and now the turn of human being. What is the guarantee that there will be no side effect. You can't compare humans to animals.<br><br>How are they going to confirm that the vaccine worked? By exposing the blood and tissues of the "vaccinated" and their un-vaccinated controls to Ebola. | Safety of vaccine- negative<br><br>Safety of vaccines- negative<br><br>Safety of vaccines- negative<br><br>Safety of vaccine component- negative | popular knowledge about vaccine side effects<br><br>Popular understanding about how to test vaccines' efficacy | **Risk society discourses**<br>• Biomedical explanations of safety<br>• Alternative explanations on "unsafety" and risk involved<br>• Alternative explanation about risks involved |

| | | | |
|---|---|---|---|
| Why are you vaccinating the citizens of this country with a vaccine you don't even know what constitutes to it!<br><br>All vaccines in use were tried anyway. Ebola vaccine trial is no different. Stop de ambiguity please. We shall avail ourselves for this trial. We trust its safety.<br><br>The most important thing is to establish whether the vaccine is safe and that has been established. It's also established that the vaccine is not going to cause any Ebola disease.<br><br>The posts show how ignorant we are as a people about vaccine trials. The journalist would have done a good job if he went further to educate us. As is typical of Ghanaian journalists he chose to sensationalize the story.<br><br>The volunteers are not being forced. It's their ignorance killing them | Safety of vaccine – positive<br><br>Safety of vaccines – positive<br><br>Ignorance about trials<br><br>Ignorance about trial procedures | Biomedical explanations for vaccine safety<br><br>Biomedical justification for public ignorance of trials<br><br>Biomedical explanation for consent processes | • Biomedical explanation of the safety of vaccine<br><br>• Biomedical interpretation of ignorance about vaccines |
